# An unusual presentation of toothpick penetration of colon

**DOI:** 10.4103/0974-2700.70773

**Published:** 2010

**Authors:** Imtiaz Wani, Shamima A Wani, Shabir Mir, Khursheed Parra

**Affiliations:** Department of Surgery, SMHS Hospital, Srinagar, Kashmir, India; 1 Department of General Medicine, SKIMS, Srinagar, India

**Keywords:** Dementia, toothpick, old age

## Abstract

This case report presents the delayed unusual presentation of plastic toothpick penetrating transverse colon 3 months after ingestion with localized peritonitis. Role of omentum “policeman of abdomen” for salvage is highlighted. Careful observation and long-term lookup for any neglected ingested foreign body are stressed. The delayed presentation can be sometimes proving as a surgical emergency.

## INTRODUCTION

**Figure 1:**
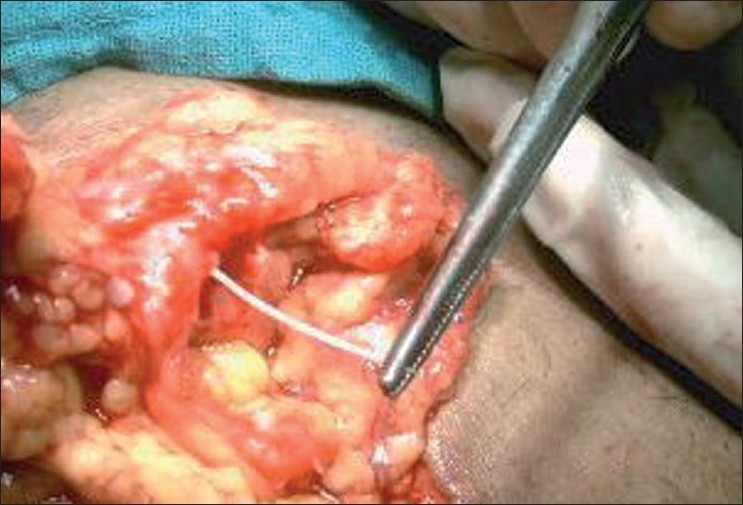
Plastic toothpick emanating from colon after penetrating its wall with inflamed heaped up omentum around it

**Figure 2:**
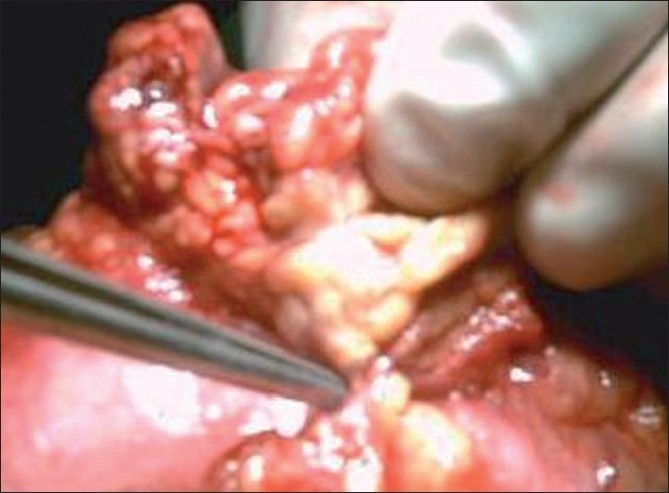
Inflammed looking omentum freed from site of penetration

An 82-year-old man, with dementia, presented to the emergency department with right lower abdominal pain, nausea, vomiting, constipation, and fever of 3 days duration. His vital signs were normal, except a temperature of 100°F. The only positive findings on his clinical examination were rebound tenderness and guarding in a right lower abdomen. Other than a leukocytosis of 10,500/mm^3^, with 88% neutrophils, his blood urea and serum creatinine were 40 mg/dL and 1.2 mg/dL, respectively. His urine analysis, metabolic profile, and serum amylase levels were normal. Computerized tomography of abdomen revealed the presence of perforated appendicitis. The patient underwent emergency laparotomy where it was noticed that the omentum was adherent to the antimesenteric border of a transverse colon in a right lower quadrant, and the appendix looked normal. Further exploration of the abnormally noticeable omentum revealed a yellow toothpick of 0.5 cm thick emanating from the organized omental lump, emerging about 5 cm from the hepatic flexure of a transverse colon [Figures 1 and 2]. The toothpick was extracted, and the patient made an uneventful recovery. Toothpicks in the gastrointestinal tract have a tendency to lodge in a location where there is an anatomic sphincter, acute angulations, physical narrowing prior surgery, or congenital gut malformation. Predisposing factors for this malady are persons with low IQ, personality disorders, those wearing artificial dentures, and alcoholics, callousness during toothpick use, palatal insensitivity, and pediatric age group. Dementia sometimes may create unawareness and loss of recollection while swallowing a toothpick which may prove challenging to reach a fast diagnosis. Ingested toothpicks are notorious for manifesting as a gastrointestinal bleeding, gut obstruction, bowel perforation, sepsis hemorrhage, and death.[1] Toothpicks have been reported to have the highest rate of impaction and perforation (9%).[2] Perforations of the gastrointestinal tract by ingested toothpicks are rare, with an annual rate of 0.2 per 100,000 persons.[3] Toothpick-related perforations are reported throughout the gastrointestinal tract, including the stomach, duodenum, small bowel, Meckel’s diverticulum, appendix, cecum, sigmoid colon, and rectum, with complications. Symptoms and signs associated with toothpick perforation mimic several intra-abdominal diseases, including diverticulitis, appendicitis, renal colic, and inflammatory bowel disease. Toothpick lodged in a colon is forced by peristalsis to make a sharp right turn, resulting in penetration of the mucosa, which can lead to migration to other organs close to the perforating site, thereby demonstrating a very different clinical pathology.[1]

Plain radiographic study usually do not identify toothpick unless being radio-opaque. Abdominal ultrasound demonstrates swallowed toothpicks frequently as a hyperechoic, thin, straight line, or a hyperechoic dot. Computed tomography scan of abdomen is used to confirm the locations of both ends of the toothpick, location of perforation, and the extent of intra-abdominal inflammation either with or without abscess formation and in excluding findings requiring surgical intervention.[4] Ingested toothpicks are hyperdense on computed tomography. Colonoscopic removal of toothpicks obviates the need for surgical intervention. Surgical intervention is done where toothpick leads to complications of peritonitis, abscesses, fistulas, migration of toothpicks to adjacent extra-colonic structures, in intractable bleeding, or those having failed endoscopic retrieval. Senile dementia and use of the toothpick could be suggestive of unintentional ingestion of toothpick. Toothpick in gut can be sometimes an intraoperative occult diagnosis, which is unexpected. Delayed manifestations may be attributed to chronic constipation in this case.
